# Disposable, Paper-Based, Inkjet-Printed Humidity and H_2_S Gas Sensor for Passive Sensing Applications

**DOI:** 10.3390/s16122073

**Published:** 2016-12-06

**Authors:** Abdul Quddious, Shuai Yang, Munawar M. Khan, Farooq A. Tahir, Atif Shamim, Khaled N. Salama, Hammad M. Cheema

**Affiliations:** 1 Research Institute for Microwave and Millimeterwave Studies (RIMMS), National University of Sciences and Technology (NUST), Islamabad 44000, Pakistan; 12mseeaquddious@seecs.edu.pk (A.Q.); 12mseemmkhan@seecs.edu.pk (M.M.K.); farooq.tahir@seecs.edu.pk (F.A.T.); 2 Computer, Electrical and Mathematical Sciences and Engineering (CEMSE), King Abdullah University of Science and Technology (KAUST), Thuwal 23955-6900, Saudi Arabia; shuai.yang@kaust.edu.sa (S.Y.); atif.shamim@kaust.edu.sa (A.S.); khaled.salama@kaust.edu.sa (K.N.S.)

**Keywords:** chipless radio frequency identification, gas sensor, hydrogen sulfide gas, inkjet printing, internet-of-things, paper substrate, wireless applications

## Abstract

An inkjet-printed, fully passive sensor capable of either humidity or gas sensing is presented herein. The sensor is composed of an interdigitated electrode, a customized printable gas sensitive ink and a specialized dipole antenna for wireless sensing. The interdigitated electrode printed on a paper substrate provides the base conductivity that varies during the sensing process. Aided by the porous nature of the substrate, a change in relative humidity from 18% to 88% decreases the electrode resistance from a few Mega-ohms to the kilo-ohm range. For gas sensing, an additional copper acetate-based customized ink is printed on top of the electrode, which, upon reaction with hydrogen sulphide gas (H_2_S) changes, both the optical and the electrical properties of the electrode. A fast response time of 3 min is achieved at room temperature for a H_2_S concentration of 10 ppm at a relative humidity (RH) of 45%. The passive wireless sensing is enabled through an antenna in which the inner loop takes care of conductivity changes in the 4–5 GHz band, whereas the outer-dipole arm is used for chipless identification in the 2–3 GHz band.

## 1. Introduction

An estimated 200 billion devices will be connected to the Internet by the year 2025 in the Internet-of-Things (IoT) realm [1]. A typical IoT system requires three ingredients: firstly, the wide variety of sensors that measure environmental, industrial, biological, activity, imaging, and other parameters; secondly, the connectivity mechanism between these sensors and their central controller; and thirdly, the plethora of applications using these two enablers in the domain of healthcare, wearables, transportation, smart homes, connected cities, and industries [2,3,4,5,6,7,8,9].

The staggering number of required sensors and their associated cost has motivated research to find ways of making them pervasive and reduce their cost to such an extent that they can potentially become disposable. To that end, radio frequency identification (RFID) technology has emerged as a strong candidate for widespread usage in the above-mentioned scenarios. In the last few years, RFID has transitioned from a mere identification technology to a versatile platform that has been used for tracking, localization, and remote sensing. In the last of these categories, RFID has been demonstrated for wireless gas and humidity sensing [10,11,12]. In addition, many similar inkjet-printed sensors, without RFID functionality, have also been presented on various substrates such as paper, polyimide, and polyethylene terephthalate (PET) [13,14,15,16,17,18]. Among these sensors, resistive response types tend to have a slow response time (~60 min) [10,11]. On the other hand, capacitive response type sensors have a faster response time (<10 min); however, their integration with antenna for remote identification has not been widely published [14,15,16,17,18,19]. Lastly, sensors utilizing special materials such as carbon nanotubes pose challenges of fabrication and can be less selective in an ambient environment [12]. 

This paper presents a dual-functionality, paper-based, inkjet-printed humidity and gas sensor. For humidity sensing, the resistive response of an interdigitated electrode, made of silver nanoparticle ink is evaluated to yield a fast response time of ~3 min. On the other hand, for hydrogen sulfide sensing, a copper acetate-based sensing material that is sensitive to H_2_S is printed over the electrode. The detection of hydrogen sulfide, due to its toxic nature, is critically important in many industrial and residential settings [20]. The sensor electrode is integrated with an antenna which is a combination of a loop and a dipole, potentially enabling wireless sensing using the frequency domain chipless RFID technique. The proposed design omits the use of discrete components and provides a low-cost alternative for humidity and gas sensing.

## 2. Sensor Design and Working Principle 

Humidity and gas sensing through printed and passive methods pose few basic requirements. For the former, a structure that is able to change its electrical properties based on ambient humidity is desirable. On the other hand, for gas sensing, a specific reagent that is sensitive to the target gas and results in a measurable electrical, physical, or optical change is needed. Lastly, a mechanism to detect and interpret these changes is required for the meaningful usage of such sensors. To that end, this paper combines three elements to achieve the above-mentioned tasks. Firstly, an interdigitated electrode, made using silver nano-particle ink combined with an underlying paper substrate, together perform as a humidity sensor. Secondly, aiming for the sensing of toxic hydrogen sulfide (H_2_S) gas, a printable chemi-resistive film of copper acetate is utilized as a reagent. Due to the chemical reaction between the reagent and H_2_S, the physical, optical, and electrical features of the electrode change in real time. The third element of the system involves the passive detection of these changes by adopting the chipless RFID technique [21]. This is achieved by placing the sensor electrode as a load to a double loop folded dipole antenna whose design will be discussed in subsequent sections.

### 2.1. Paper Substrate

The choice of substrate for inkjet printing of the electrode, reagent, and the antenna requires careful consideration of parameters such as porosity, surface roughness, thickness, permittivity, and cost. Organic flexible substrates such as paper and plastic are regularly being used to design antennas, sensors, and chipless RFID tags [10,11,12,13,21,22,23,24,25,26]. More specifically, paper, due to it lower cost and scalability to roll-to-roll and reel-to-reel printing has become a preferable choice for inkjet printing. One downside associated with a paper substrate, especially if it is of porous nature, is the inferior printing resolution, as the conductive ink during the printing process seeps into adjacent pores, leading to deformation in the printed designs. To avoid this issue, Kodak photo paper is utilized in this work which is glossy and flexible with low porosity. The selected paper has a permittivity, loss tangent, and thickness of 2.6, 0.05, and 0.28 mm respectively. 

### 2.2. Interdigitated Electrode

**Figure 1:**
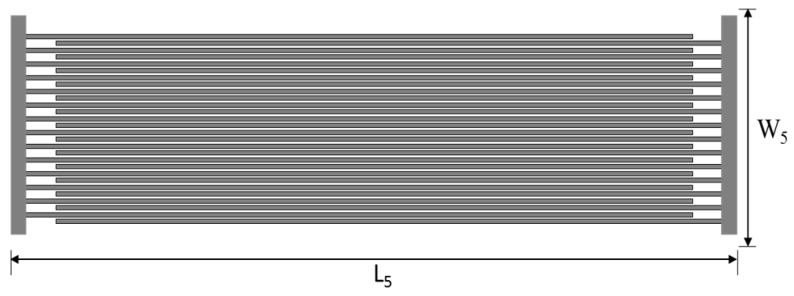
Layout of the proposed sensor’s interdigitated electrode. L_5_ = 45.75 mm and W_5_ = 20 mm.

The layout of the sensor’s interdigitated electrode is shown in Figure 1. With dimensions of 45.75 × 20 mm^2^, the electrode is connected to the inner loop of the antenna at its edges and acts as the base for printing of the chemi-resistive layer. Each electrode includes a total of 14 fingers with lengths and widths of 40 mm and 200 μm, respectively. The spacing between the adjacent electrode fingers is also 200 μm. These dimensions are optimized after a number of printing iterations to achieve optimum fabrication results. The electrode is inkjet-printed using Dimatix DMP 2831 printer using silver nano-particle ink from UT Dots on the Kodak photo paper substrate discussed above. The drop spacing for the nano-particle ink (silver 20% by weight) is 25 μm with a nozzle size of 10 pL. After printing 5 layers of the conductive ink on top of each other, the electrode is heat sintered at 120 °C for 10 min to achieve a conductivity of 6 × 10^6^ S/m with a conductor height of ~2 μm. Post-sintering resistance measurements show a high initial resistance of 10 MΩ.

### 2.3. Chemi-Resistive Ink

After preparation of the electrode, a mechanism for gas sensing is devised. For this purpose, chemicals that are sensitive to H_2_S and are printable using the inkjet printing process are investigated. The latter requirement is critical, as the specific viscosity and surface tension is required for accurate printing on the paper substrate. The printable chemical ink when exposed to gas leads to a visual change in its color as well as variation in its chemical properties. The latter is responsible for the change in the resistivity of the ink, hence the name “chemi-resistive layer”. 

Hydrogen sulfide gas is known to react with metal acetates that typically result in a formation of metal sulfides. The electrical response of copper acetate films in the presence of hydrogen sulfide gas has been reported [13,23] and is governed by the following chemical equation:

(1) $$\mathrm{Cu}{\left({\mathrm{CH}}_{3}\mathrm{COO}\right)}_{2}\left(s\right)+H_{2}S\left(g\right)\overset{\mathrm{yields}}{\to }\mathrm{CuS}+2{\mathrm{CH}}_{3}\mathrm{COOH}$$

The original chemi-resistive ink of copper acetate has low conductivity; however, in the presence of H_2_S, copper sulfide is formed, increasing the conductivity [27]. This change in turn affects the electrode’s conductivity and the antenna’s behavior as the electrode acts as a load to the antenna. 

The composition of the copper-acetate solution is critical for successful printing using the inkjet printer. This is achieved by mixing metal salt in three solvents namely water, ethylene glycol (EG), and iso-propanol (IPA). A volume ratio of 8:1:1 is used for a water/EG/IPA solvent mixture for a copper-acetate solution with a final concentration of 0.1 mol. For inkjet printing of the chemi-resistive film, three layers are printed on top of the interdigitated electrode with a drop spacing of 30 μm. This tag is again heat sintered at a temperature of 150 °C for 15 min before it is used for gas sensing.

## 3. Antenna Design

**Figure 2:**
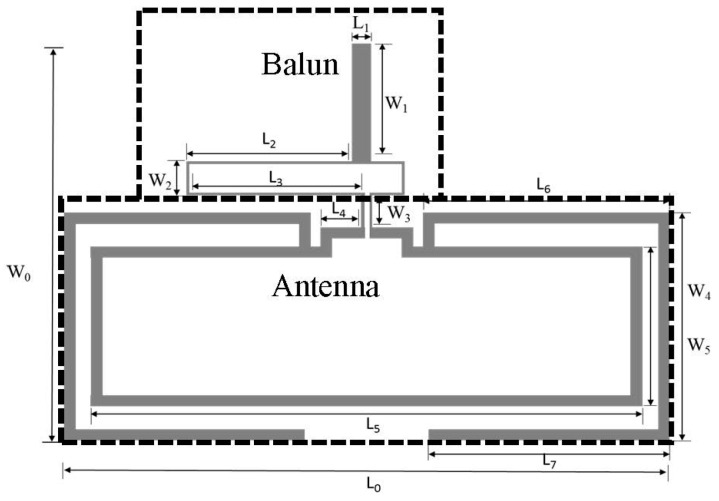
Layout of the proposed antenna fed using a balun. The wide space in the center is left intentionally for sensor integration.

**Figure 3:**
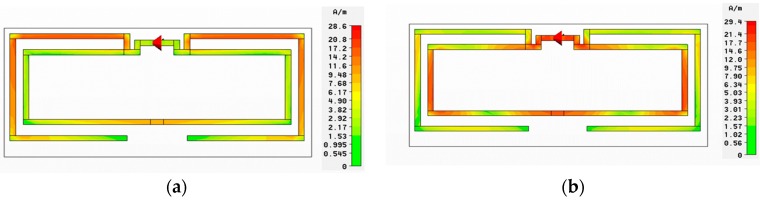
Current distribution in the two loops of the antenna. (**a**) Outer dipole at 2.8 GHz; (**b**) Inner loop at 4.6 GHz.

The specialized antenna designed in this work plays two distinct roles (Figure 2). Firstly, the inner loop connects and encircles the interdigitated electrode that acts as its load. Any change in the electrode’s properties manifests as a change in frequency behavior of the inner loop. The second role of the antenna is to achieve a unique identification for each specific tag. This is realized by altering the dimensions of the outer dipole arms of the antenna. Using this chipless RFID approach, wherein each tag has a unique electromagnetic signature, passive sensing through its radar cross-section (RCS) can thus be accomplished [21]. Based on these requirements, the outer identification dipole is designed to resonate in the 2–3 GHz range, whereas the inner sensing loop resonates between 5 and 6 GHz as verified by the current distribution shown in Figure 3. The frequency and bandwidth are controlled by the dipole length, horizontal arm lengths L_5_, L_6_, and L_7_, and vertical arm lengths W_4_ and W_5_. The antenna is simulated in CST Microwave Studio (ver. 14), and its optimized parameters are shown in Table 1. The horizontal arm lengths L_6_ and L_7_ control the resonance frequency in the identification band. For instance, increasing L_6_ and L_7_ decreases the resonating frequency while maintaining the operational bandwidth due to constant vertical arm lengths.

**Figure 4:**
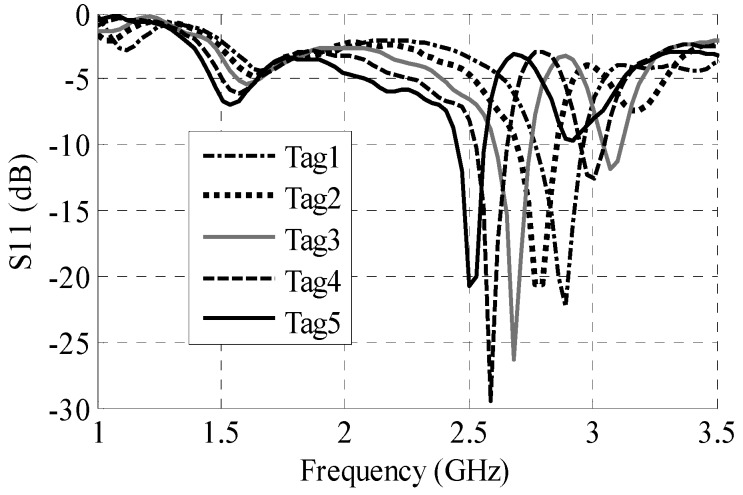
Antenna response for unique identification.

The 2.8 GHz frequency band due to the outer dipole is utilized for the tag’s chipless identification. This is achieved by creating minor changes in the dimensions L_6_ and L_7_, thereby getting varied resonances between 2.7 GHz and 2.9 GHz. Table 2 enlists dimensions of five such example tags, whereas preliminary simulation results (Figure 4) show the corresponding unique resonances. Following the same procedure and utilizing frequency shift encoding technique as presented in [21], a large number of individual tags can thus be generated.

In order to enable characterization of the antenna via an SMA connector, a balun is also included in the design. The balun introduces a 180° phase shift between the two microstrip lines that differentially excite the dipole antenna. To obtain this phase shift, the difference in the microstrip lengths at the center frequency is approximately λ2. The balun is realized on the same layer as the antenna, whereas its partial ground plane is printed on a separate layer and subsequently glued to the antenna layer.

## 4. Experimental Results

**Figure 5:**
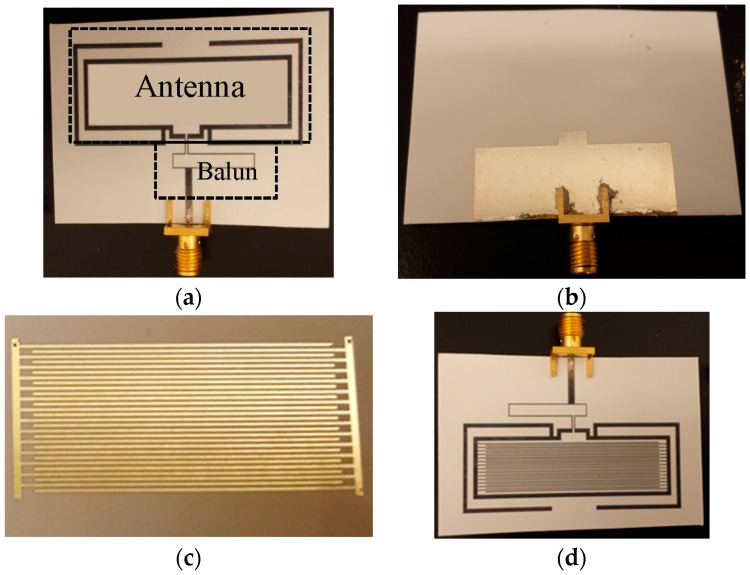
Fabricated prototypes. (**a**) Top view of antenna; (**b**) Bottom view of antenna; (**c**) Inkjet-printed interdigitated electrode; (**d**) Complete prototype with antenna, balun, and interdigitated electrode.

The interdigitated electrode, dipole antenna, and the chemi-resistive film are all fabricated using inkjet printing as discussed earlier through individually optimized steps for each [21]. The fabricated prototypes are shown in Figure 5. An SMA connector is mounted using conductive silver epoxy and an Agilent VNA (Model: N5232A) is used for s-parameter measurements. 

**Figure 6:**
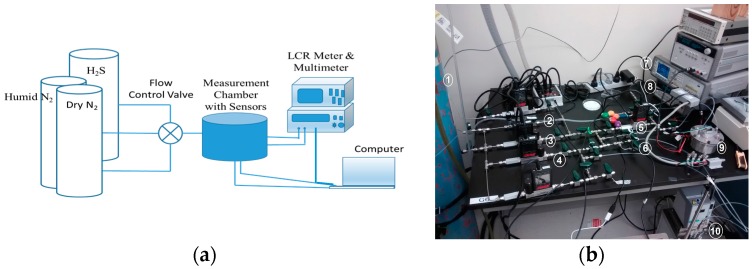
Measurement setup for humidity and gas sensing. (**a**) Block diagram; (**b**) Laboratory setup. 1. Gas cylinder. 2. MFC (Mass Flow Controller) connected to H_2_S gas. 3. MFC connected to dry Nitrogen. 4. Gas flow through this MFC going to bubbler. 5. MFM (Mass Flow Meter). 6. Connected to bubbler for humidity measurement. 7. LCR meter. 8. Multimeter. 9. Measurement chamber with gas inlet and gas outlet; test leads connected to LCR and Multimeter, contains sensor tag. 10. Bubbler to provide humidity.

The experimental setup for conductivity measurements under controlled H_2_S exposure is shown in Figure 6. The details of the setup are discussed in [28,29]. The system consists of the following:

- A gas line with a flow control valve coming from the H_2_S gas cylinder with controllable concentration. The Mass Flow Controller (MFC) is provided by Alicat Scientific Inc. (Tucson, AZ, USA).
- An aluminum chamber for housing the sensor with two leads for external connections. In addition, the chamber consists of commercial humidity sensor (Honeywell HIH-4000-003) for calibration purposes.
- The chamber is connected to an LCR meter (Agilent E4980A) that is accessed by a PC through RS-232 to USB interface and National Instruments (NI) LabVIEW software (ver. 2011).

### 4.1. Antenna Characterization

**Figure 7:**
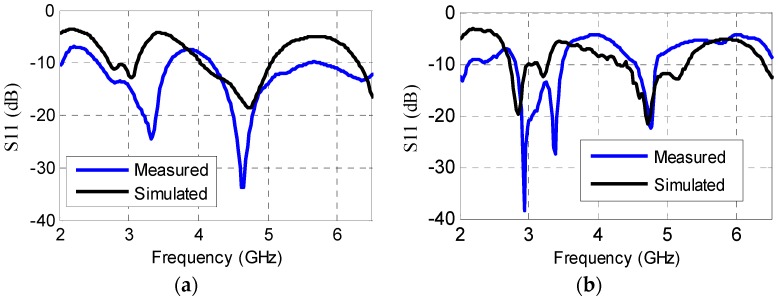
Simulated and measured results on Kodak photo paper (**a**) Return loss of antenna only; (**b**) Return loss of antenna with electrode on Kodak photo paper.

The dipole antenna is fabricated on a paper substrate. Figure 7a shows adequate agreement between simulated and measured results with two distinct bands appearing at 2.8 GHz and 4.6 GHz. As only one side of this paper is glossy, two individual layers are used, first to print antenna and interdigitated electrode and second to print the partial ground plane. The two layers are subsequently glued together, thus increasing the thickness from 0.280 mm to 0.560 mm. In Figure 7b, the simulated and measured return loss of the antenna with an interdigitated electrode is shown. The slight shift between the simulated and measured results is attributed to the glue used between the two layers of photo paper.

### 4.2. Humidity Sensing

**Figure 8:**
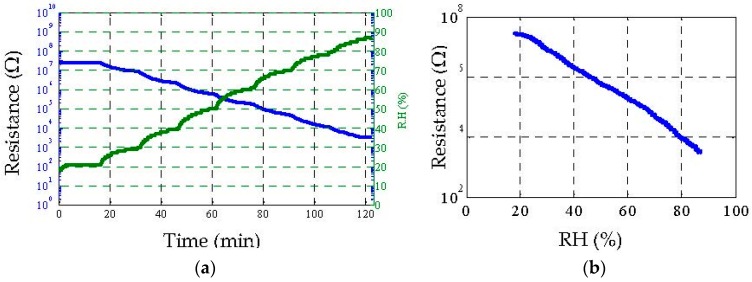
Humidity sensing results. (**a**) Measured resistance (Blue) of sensor for different humidity levels (Green) with respect to time; (**b**) Measured resistance of sensor with respect to varying humidity only.

Utilizing the measurement setup of Figure 6, the humidity sensing capability of the sensor was analyzed. The bare interdigitated electrode without the chemi-resistive ink was placed in the chamber, and the relative humidity (RH) was increased with time. As the paper substrate has a tendency to absorb moisture, the conductivity of the electrode was expected to increase due to initiation of ion conduction as explained in [26]. This was verified, as shown in Figure 8a,b, by a decrease in resistance from 10 MΩ to a few tens of kΩ as the RH was increased from 18% to 88%. The decrease in resistance, measured through the LCR meter, started at about the 20 min mark and continued to decrease linearly as RH was increased.

### 4.3. Gas Sensing

**Figure 9:**
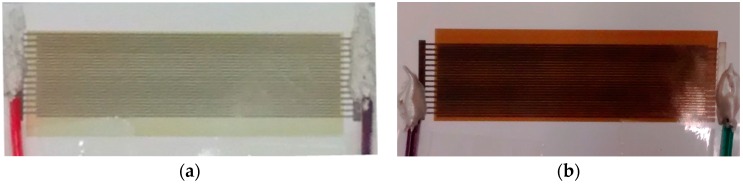
Inkjet-printed sensor module (**a**) before exposure; and (**b**) after exposure to H_2_S.

**Figure 10:**
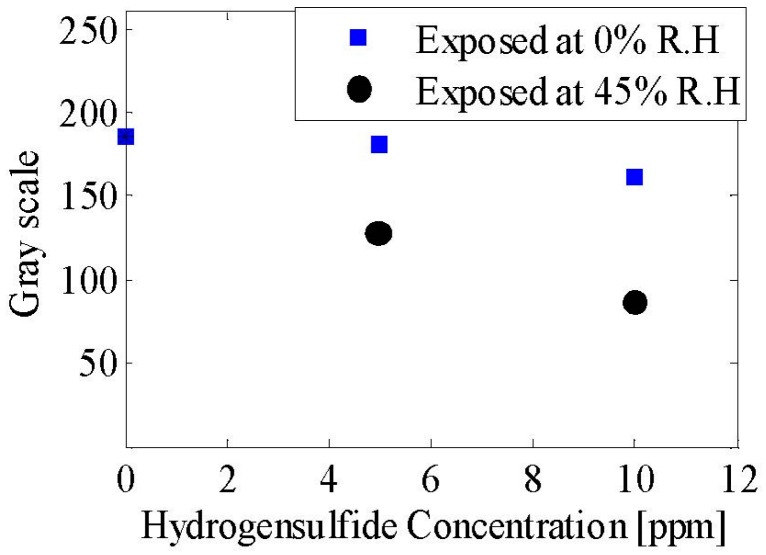
Optical response of the sensor with different concentrations of H_2_S and relative humidity.

After the antenna and humidity measurements, the copper-acetate solution was inkjet-printed on the tags and subsequently exposed to H_2_S concentrations of 5 ppm and 10 ppm for 100 min without humidity (0% RH) and with humidity (45% RH). All the experiments discussed in this paper were performed at room temperature. The response of the sensor was assessed in two ways: the optical response and the electrical response. In the former, the exposure to H_2_S changes the greenish-blue color of the copper acetate to a darker shade due to the formation and growth of copper sulfide particles, Cu_2_S and CuS, whose characteristic color is dark grey and indigo blue, respectively. Exposure to a 10 ppm concentration of H_2_S changes the color to dark brown/blackish green. The un-exposed and exposed samples of the sensor module are shown in Figure 9, verifying that the sensor can be used as a simple optical indicator for the presence of H_2_S gas. The exact color depends on the humidity, exposure time, and gas concentration. It is important to note that the change in color is an irreversible process making the sensor suitable for a one-time use only. This disposability characteristic is justified due to the sensor’s low cost of fabrication when scaled from lab-scale inkjet printing to mass scale roll-to-roll and reel-to-reel printing. To represent the optical response in a meaningful way, a graph with a color scale from 0 to 255, where 0 corresponds to black and 255 corresponds to white color. Shown in Figure 10, as the H_2_S concentration is increased from 0 ppm to 10 ppm, the scale reading decreases. Furthermore, as the humidity is increased to 45%, the scale reading decreases further as high humidity improves the sensitivity of the chemi-resistive layer; hence, more copper sulfide particles are formed on the sensor surface, leading to a decrease in color scale reading.

**Figure 11:**
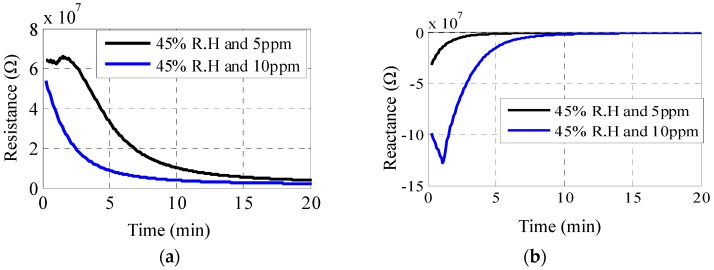
Change in (**a**) real impedance; and (**b**) reactance of sensor exposed to 5 ppm and 10 ppm concentrations of H_2_S at a relative humidity of 45% and measured at 2 MHz.

**Figure 12:**
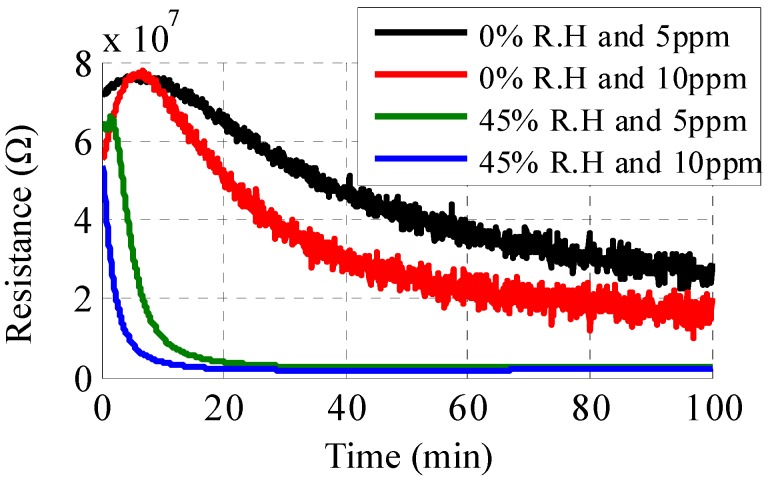
Comparison of change in sensor’s real impedance exposed to 5 ppm and 10 ppm concentration of H_2_S at 0% and 45% RH.

In addition to the optical changes, the electrical properties of the sensor also change under exposure to H_2_S gas. This happens due to the formation of Cu_2_S particles on top of the electrode fingers, thus forming a conductive path resulting in a decrease in resistance. At the particle level, this increase in conductivity is attributed to the percolation in the continuous conducting networks and tunneling between the isolated conducting particles [30,31]. Figure 11a shows the resistance variation of the sensor exposed to concentrations of 5 and 10 ppm H_2_S at a relative humidity of 45%. A sharp decrease is observed in the initial few minutes, after which both curves saturate. The response time, which is defined as the time required to attain a 90% change of the total resistance change, is 3 min for a 10 ppm concentration, as compared to 7 min for 5 ppm. This is a consequence of a faster chemical reaction and a formation of copper sulfide particles. Similar behavior is observed for the reactive part of the sensor, as shown in Figure 11b. Figure 12 illustrates the dependence of the sensor response on two different levels of humidity. An inverse relationship is observed between humidity and response time. More specifically, response time increases from being less than 10 min to more than 60 min as humidity decreases from 45% RH to 0% RH. Nevertheless, for typical humidity levels of 30%, response time can be extrapolated to around 10 min. Furthermore, as the variation in the sensor’s resistance is different for humidity and H_2_S, and optical changes also happen for the latter, differentiation between humidity and gas sensing can be achieved. 

**Figure 13:**
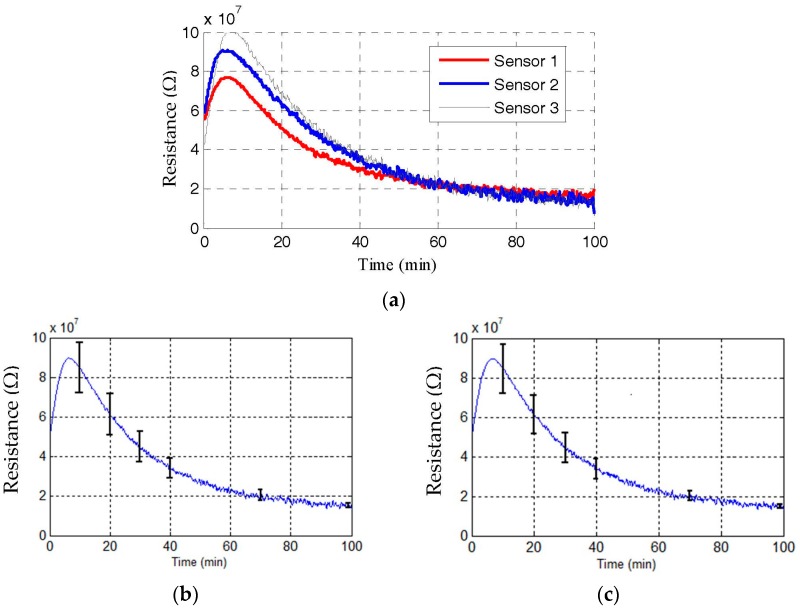
(**a**) Response of three sensors to 0% RH and 5 ppm H_2_S concentration; (**b**) Average deviation between three sensor responses; (**c**) Maximum deviation between three sensor responses.

The repeatability of sensor performance was verified by fabricating and characterizing three samples through the same process. Shown in Figure 13a, the response of these three sensors, under 0% RH and 5 ppm H_2_S gas exposure, shows a very close match in the steady state. Using the mean resistance values from the three sensors for each time instant, the deviation of each sensor response from the mean was calculated, which was subsequently used to calculate the average and maximum deviation. These are plotted in Figure 13b,c, respectively, alongside the mean response. Slightly higher spread is observed during the initial transient period, whereas response closely matches the steady state.

**Figure 14:**
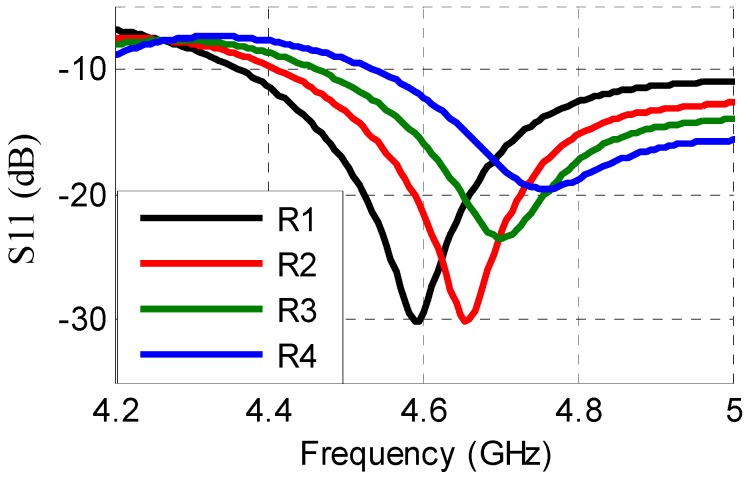
Return loss of antenna integrated with sensor with conductivity values at different time instances obtained from experimental data. R1 = 280 Ω, R2 = 200 Ω, R3 = 166 Ω, and R4 = 136 Ω.

In order to validate the overall operation of the sensor including the antenna, the change in conductivity of the sensor is included in a full-wave 3D post-measurement simulation. This method is adopted, as it is not possible to measure the s-parameters, while the tags are placed in the gas measurement chamber. Thus, as shown in Figure 14, four measured resistance values of the electrode are used in the simulation model of CST Microwave Studio, yielding four distinct frequencies of the chipless RFID tag. This emulates how the sensor along with the antenna would behave in reality for varying concentrations of H_2_S gas. These frequencies when passively sensed can be directly related to the level of H_2_S concentration, thus verifying the overall operation.

A detailed comparison with published works is presented in Table 3. It is interesting to note that, due to the electrode structure used, the presented work shows the fastest response time of 3 min among resistive type sensors [10,11,12]. Furthermore, it is the only gas sensor among the reported fully-printed sensors that not only has wireless sensing capability but also identification functionality [14,15,16,17,18]. Lastly, compared to [13], in which H_2_S sensing is offered in a smaller tag dimension, the presented work demonstrates a sixfold lower response time along with the antenna integration for wireless identification. 

## 5. Conclusions

A dual-functionality, paper-based, inkjet-printed humidity and gas sensor has been presented. The complete sensor consists of an interdigitated electrode, a copper acetate-based chemi-resistive ink, and a specialized loop-dipole antenna combination to enable passive sensing. The miniaturized electrode results in a fast response time of ~3 min, which is the best among resistive type sensors. The gas sensing behavior has been characterized both electrically and optically. The novel use of the antenna’s inner loop for the wireless monitoring of the sensor’s resistance and outer loop for chipless identification makes it a promising choice as a low-cost pervasive humidity and gas sensor. 

## Figures and Tables

**Table 1:** Detailed dimensions of proposed antenna.

Parameter	Dimension	Parameter	Dimension
L_0_	50.25 mm	W_0_	35 mm
L_1_	1.64 mm	W_1_	10.75 mm
L_2_	13.75 mm	W_2_	3.00 mm
L_3_	14.57 mm	W_3_	2.75 mm
L_4_	3.5 mm	W_4_	14 mm
L_5_	45.75 mm	W_5_	20 mm
L_6_	20.5 mm	L_7_	20 mm

**Table 2:** L_6_ and L_7_ dimensions for unique identification.

	L_6_	L_7_
Tag 1	20.5 mm	20 mm
Tag 2	21.5 mm	21 mm
Tag 3	22.5 mm	22 mm
Tag 4	23.5 mm	23 mm
Tag 5	24.5 mm	24 mm

**Table 3:** Comparison with published works.

Reference	Sensor Type	Tag Size	Response Time	Substrate	Integrated with Antenna	Identification Capability
[10]	Humidity, Resistive type	2.9 mm × 0.6 mm	More than 60 min	Paper	No	No
[11]	Humidity, Resistive type	188 mm × 9 mm	20–100 min	Paper	Yes	No
[12]	Gas (Ammonia & Nitrogen Oxide), Resistive type	118 mm × 27 mm	-	Paper	Yes	No
[13]	Gas (Hydrogen Sulfide), Resistive type	20 mm × 11 mm	Approx. 20 min	Paper	No	No
[14]	Humidity, Capacitive type	Sensor area is 200 mm^2^	4–11 min	Polyimide and Polyethersulphone	No	No
[15]	Humidity, Capacitive type	12 mm × 8 mm	Approx. 24 s	PET	No	No
[16]	Humidity, Capacitive type	6.3 mm × 1.85 mm	5–6 min	Polyimide	No	No
[17]	Humidity, With both resistive and capacitive measurements	5.4 mm × 5.4 mm	Less than 20 s	Polyimide	No	No
[18]	Humidity, Capacitive type	12 mm × 12 mm	Approx. 20 min	PET	No	No
This work	Dual functionality Humidity & Gas (Hydrogen Sulfide), Resistive type	45.75 mm × 20 mm	3 min	Paper	Yes	Yes
